# Supplementary material for: Experimental Prototype of Electromagnetic Emissions for Biotechnological Research: Monitoring Cocoa Bean Fermentation Parameters
Source: Foods. 2023 Jun 29;12(13):2539. doi: 10.3390/foods12132539 (PMC10340622; doi:10.3390/foods12132539)
Supplement: Supplementary file 1 [file foods-12-02539-s001.zip › foods-2440718-supplementary-revised.pdf]

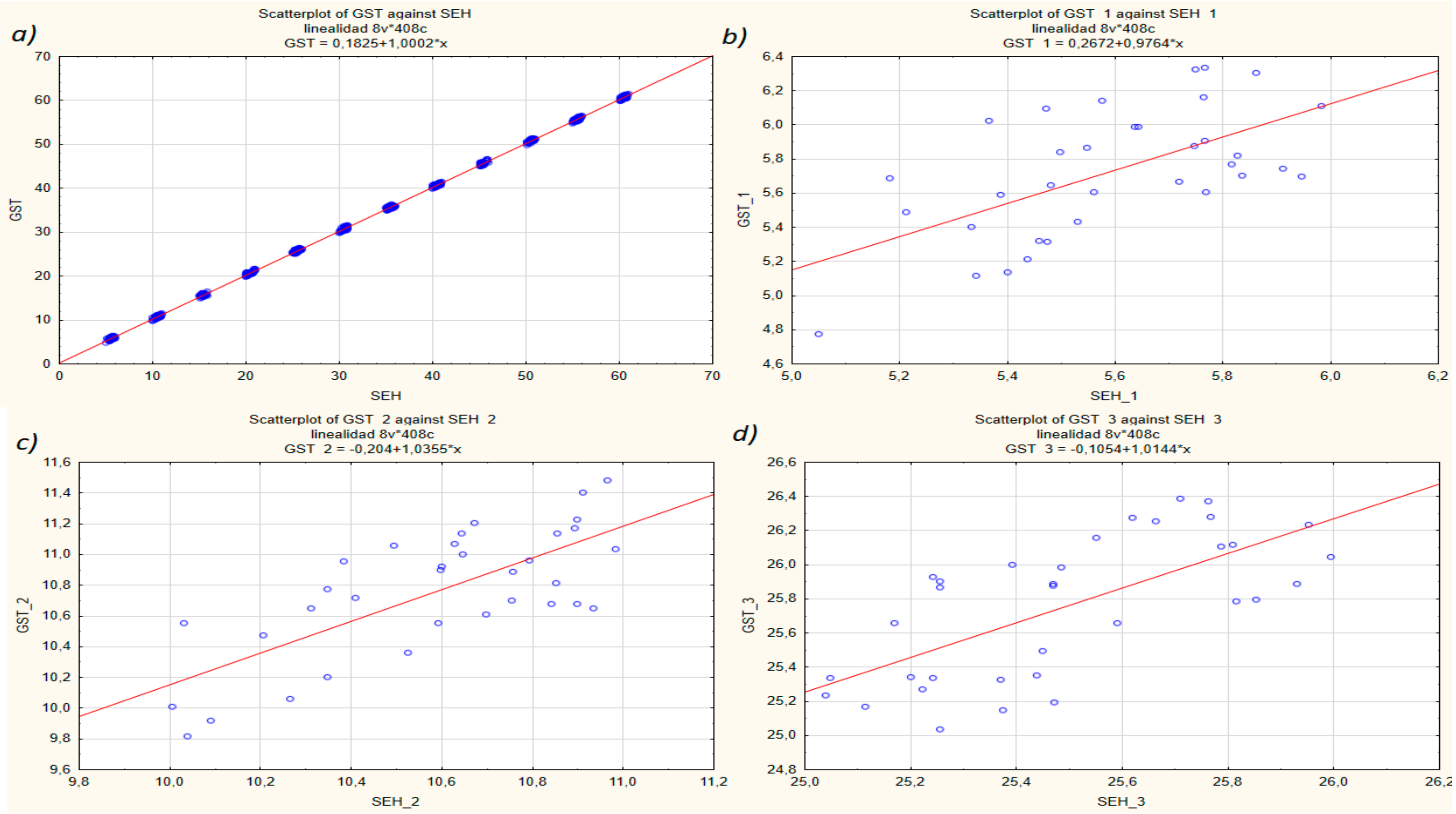

Figure S1 Linearity graph. Vertical axis GST horizontal axis SEH in different OMF density (a), cloud of points (4-7mT) (b), cloud of points (9-12mT) (c), cloud of points (25-27mT) (d).

Table S1. Experimental Design. Factors, levels, and Responses R1 OMF density (6A) levels: analyst1…analyst7; R2 OMF: density (30A) levels: day 1…day 7; R3: OMF density (90A) levels 20, 25, 30, 35, 40, 45, 50

Factor, levels responses	Units	Type	SubType	Minimum	Maximum	Coded Low	Coded High	Mean	Std. Dev.
Radial points ρ	m	Numeric	Continuous	-0.5045	0.5045	-1 ↔ -0.30	+1 ↔ 0.30	0.000	0.2776
Radial points α	m	Numeric	Continuous	-0.5045	0.5045	-1 ↔ -0.30	+1 ↔ 0.30	0.000	0.2776
Axial points Z	m	Numeric	Continuous	-0.1534	0.6034	-1 ↔ 0.00	+1 ↔ 0.45	0.225	0.2082
Levels	-	Categorical	Nominal	Level 1	Level 7	-	-	-	7.00
OMF density (6A)	mT	Numeric	Continuous	0.00078	5.97101	-	-	2.13	2.04
OMF density (30A)	mT	Numeric	Continuous	0.00531	29.9567	-	-	10.76	10.27
OMF density (90A)	mT	Numeric	Continuous	0.00342	89.6288	-	-	31.93	30.15

Table S2 The ANOVA of the OMF Density at different intensities (R1, R2, and R3). There are statistically significant differences in the radial coordinates of the prototype, with a confidence of 95.0%. In all responses, the model F value of the model was significant, and the lack of fit was not significant relative to the

	Model R1					***Model R2***					***Model R1***				
Source	***Sum of Squares***	***df***	***Mean Square***	***F-value***	***p-value***	***Sum of Squares***	***df***	***Mean Square***	***F-value***	***p-value***	***Sum of Squares***	***df***	***Mean Square***	***F-value***	***p-value***
**Models**	1372.72	33	41.60	537.71	< 0.0001	33856.14	69	490.67	87.63	< 0.0001	2.901E+05	33	8791.88	185.58	< 0.0001
a z	0.5379	1	0.5379	6.95	0.0088	-	-	-	-	-	-	-	-	-	-
p	-	-	-	-	-	-	-	-	-	-	164.67	1	164.67	3.54	164.67
z	-	-	-	-	-	-	-	-	-	-	361.02	1	361.02	7.75	0.0057
A^2	748.32	1	748.32	9673.23	< 0.0001	18180.51	1	18180.51	3246.92	< 0.0001	1.568E+05	1	1.568E+05	3310.49	< 0.0001
B^2	748.30	1	748.30	9672.92	< 0.0001	18826.21	1	18826.21	3362.24	< 0.0001	1.624E+05	1	1.624E+05	3428.70	< 0.0001
C^2	0.6263	1	0.6263	8.10	0.0047	36.53	1	36.53	6.52	0.0112	315.88	1	315.88	6.67	0.0103
**Residual**	23.36	302	0.0774			1489.42	266	5.60			14307.06	302	47.37		
Lack of Fit	4.04	71	0.0568	0.6795	0.9717	240.19	35	6.86	1.27	0.1547	3512.97	71	49.48	1.06	0.3696
Pure Error	19.33	231	0.0837			1249.22	231	5.41			10794.09	231	46.73		
**Cor Total**	1396.08	335				35345.56	335				3.044E+05	335			
**Equations**	R1=79.5189 + -2.5258 * A + 0.820197 * B + 16.313 * C + -315.474 * A^2 + -321.057 * B^2 + -25.1701 * C^2					R2=27.2235 + -0.0341274 * A + 0.419543 * B + 4.67432 * C + -107.411 * A^2 + -109.302 * B^2 + -8.55944 * C^2					R3=81.9151 + -0.757741 * A + 0.246059 * B + 1.12196 * C + -28.3927 * A^2 + -28.8951 * B^2 + -1.27424 * C^2				

pure error. The equations in terms of the coded factors describe the decrease in density as a function of distance

Table S3. Confirmation. OMF Density (R1, R2, and R3) in three different working area coordinates.

Confidence = 95%

Coord. Analysis (ρ, α, z)	Responses	Predicted Mean	Observed Mean	Std Dev	n	SE Pred	95% PI low	95% PI high
(0.0, 0.0, 0.27)	R1	5.60227	5.45714	0.278137	7	0.119541	5.36703	5.83751
(-0.10,0.18,0.22)	R1	4.60613	4.77286	0.278137	7	0.12121	4.77286	4.84466
(-0.17,-0.14,0.40)	R1	4.45485	4.45485	0.278137	7	0.124951	4.20897	4.70074
(0.0, 0.0, 0.27)	R2	27.8616	27.1271	2.38681	7	1.02351	25.8479	29.8753
(-0.17,-0.14,0.40)	R2	22.1885	22.1885	2.36629	7	1.19226	19.8411	24.536
(-0.10,0.18,0.22)	R2	23.5323	25.9514	2.36629	7	1.23567	25.9514	25.9652
(0.0, 0.0, 0.27)	R3	82.0886	79.8743	6.82311	7	2.78019	76.6194	87.5577
(-0.10,0.18,0.22)	R3	69.2391	75.0271	6.88291	7	2.99953	75.0271	75.1417
(-0.17,-0.14,0.40)	R3	66.5803	66.5803	6.88291	7	3.0921	60.4955	72.6651

Table S4. ANOVA models for Temperature, pH, Humidity, and Brix responses. The models are significant as indicated by their respective F-values of 94.26, 41.34, 38.69, and 74.56. The probability of obtaining such large F-values due to noise is very low (0.01%). The p-values less than 0.0500 for model terms A, B, and AB indicate that they are significant in all four models. Values greater than 0.1000 suggest that model terms are not significant, and their removal may improve the model if there are many insignificant terms.

Source	Sum of Squares	df	Mean Square	F-value	p-value	
**Temperature model**	6666.90	31	215.06	94.26	< 0.0001	significant
A-OMF density	817.54	3	272.51	119.45	< 0.0001	
B-Time	5542.19	7	791.74	347.03	< 0.0001	
AB	307.16	21	14.63	6.41	< 0.0001	
**Pure Error**	146.01	64	2.28			
**Cor Total**	6812.91	95				
**pH model**	18.09	31	0.5836	41.34	< 0.0001	significant
A-OMF density	1.91	3	0.6359	45.04	< 0.0001	
B-Time	14.92	7	2.13	151.00	< 0.0001	
AB	1.26	21	0.0600	4.25	< 0.0001	
**Pure Error**	0.9036	64	0.0141			
**Cor Total**	19.00	95				
**Humidity model**	1207.09	31	38.94	38.69	< 0.0001	significant
A-OMF density	131.32	3	43.77	43.50	< 0.0001	
B-Time	1010.94	7	144.42	143.50	< 0.0001	
AB	64.82	21	3.09	3.07	0.0003	
**Pure Error**	64.41	64	1.01			
**Cor Total**	1271.50	95				
**Brix model**	1315.86	31	42.45	74.56	< 0.0001	significant
A-OMF density	44.80	3	14.93	26.23	< 0.0001	
B-Time	1200.66	7	171.52	301.30	< 0.0001	
AB	70.40	21	3.35	5.89	< 0.0001	
**Pure Error**	36.43	64	0.5693			
**Cor Total**	1352.29	95				

Table S5. The ANOVA for Wi, Fi, and Di responses. The models for Wi, Fi, and Di are significant. The F-values for Wi, Fi, and Di are 77.37, 31.60, and 9.59, respectively, indicating that the models are highly significant with only a 0.01% chance that the observed F-values are due to noise. The p-values for the model terms A, B, and AB are less than 0.0500 for Wi and Fi, and B is significant for Di, indicating that these terms have a significant effect on the response variable. Poisson Regression (Type III) for Fd. The significant model terms A, B, and AB, with p-values less than 0.0500. Values greater than 0.1000 indicate that the model terms are not significant. The analysis used χ^2 Log Likelihood Ratio p-values. Analysis was performed with a log link and inverse link of exp, using Maximum Likelihood (ML) analysis

Source	Sum of Squares	df	Mean Square	F-value	p-value	
**Wi model**	2911.40	31	93.92	77.37	< 0.0001	significant
A-OMF density	125.63	3	41.88	34.50	< 0.0001	
B-Time	2607.83	7	372.55	306.92	< 0.0001	
AB	177.95	21	8.47	6.98	< 0.0001	
**Pure Error**	77.68	64	1.21			
**Cor Total**	2989.09	95				
**Fi model**	42.77	31	1.38	31.60	< 0.0001	significant
A-OMF density	1.78	3	0.5937	13.60	< 0.0001	
B-Time	39.89	7	5.70	130.52	< 0.0001	
AB	1.09	21	0.0521	1.19	0.2870	
**Pure Error**	2.79	64	0.0437			
**Cor Total**	45.56	95				
**Di model**	166.54	31	5.37	9.59	< 0.0001	significant
A-OMF density	1.74	3	0.5803	1.04	0.3827	
B-Time	154.73	7	22.10	39.47	< 0.0001	
AB	10.06	21	0.4792	0.8557	0.6440	
**Pure Error**	35.84	64	0.5600			
**Cor Total**	202.38	95				

Source	χ^2	df	p-value
**Fd model**	3306.74	3	< 0.0001
A-Densidad CMO (mT)	58.69	3	< 0.0001
B-B	3020.86	2	< 0.0001
AB	401.81	6	< 0.0001

Table S6. ANOVA models for LAB, AAB, and Y responses. The models are significant, as indicated by their respective F-values of 6.49, 33.26, and 8.91. The probability of obtaining such large F-values due to noise is very low (0.01%). The p-values less than 0.0500 for model terms A, B, and AB indicate that they are significant in all three models. ANOVA Y model terms B and AB are significant. Values greater than 0.1000 suggest that, the three models provide significant results

Source	Sum of Squares	df	Mean Square	F-value	p-value	
**LAB model**	90.89	31	2.93	6.49	< 0.0001	significant
A-OMF density	32.91	3	10.97	24.27	< 0.0001	
B-Time	18.02	7	2.57	5.70	< 0.0001	
AB	39.96	21	1.90	4.21	< 0.0001	
**Pure Error**	28.92	64	0.4519			
**Cor Total**	119.81	95				
**AAB model**	320.89	31	10.35	33.26	< 0.0001	significant
A-OMF density	96.80	3	32.27	103.67	< 0.0001	
B-Time	176.00	7	25.14	80.78	< 0.0001	
AB	48.09	21	2.29	7.36	< 0.0001	
**Pure Error**	19.92	64	0.3112			
**Cor Total**	340.81	95				
**Y model**	141.92	31	4.58	8.91	< 0.0001	significant
A-OMF density	1.00	3	0.3346	0.6515	0.5849	
B-Time	119.81	7	17.12	33.32	< 0.0001	
AB	21.11	21	1.01	1.96	0.0211	
**Pure Error**	32.87	64	0.5136			
**Cor Total**	174.79	95				
